# Identification of genes involved in alcohol consumption and cigarettes smoking

**DOI:** 10.1186/1471-2156-6-S1-S112

**Published:** 2005-12-30

**Authors:** Mariza de Andrade, Curtis L Olswold, Joshua P Slusser, Larry A Tordsen, Elizabeth J Atkinson, Kari G Rabe, Susan L Slager

**Affiliations:** 1 Division of Biostatistics, Department of Health Sciences Research, Mayo Clinic College of Medicine, Rochester, MN, USA

## Abstract

We compared the results of quantitative linkage analysis using single-nucleotide polymorphisms and microsatellite markers and introduced a new screening test for multivariate quantitative linkage analysis using the Collaborative Study on the Genetics of Alcoholism data. We analyzed 115 extended non-Hispanic White families and tested for linkage using two phenotypes: the maximum number of drinks in a 24-hour period and the number of packs smoked per day for one year. Our results showed that the linkage signal increased using single-nucleotide polymorphisms compared with microsatellite markers and that the screening test gave similar results to that of the bivariate analysis, suggesting its potential use in reducing overall analysis time.

## Background

The Collaborative Study on the Genetics of Alcoholism (COGA) is a multicenter research program to detect and map susceptibility genes for alcohol dependence and related phenotypes. Numerous behavior measures were collected, two of which we considered for our study. The first is the maximum number of drinks in a 24 hour period (drink24), which can be considered a surrogate to alcoholism diagnosis and provides a quantitative measure to grade non-alcoholic individuals [1]. The second measure is the number of packs smoked per day for one year (pakyrs). Since pakyrs is highly correlated with alcohol consumption [2], these two measures are good candidates for multivariate linkage analysis. The goals of our analysis were two-fold. First, we investigated the performance of a genome-wide scan using single-nucleotide polymorphisms (SNPs) relative to the microsatellite markers. Several studies have shown gains in information when SNPs are used for qualitative traits, but advantages and disadvantages of SNPs have not been explored with quantitative traits [3,4]. Second, we evaluated a new screening test for multivariate quantitative linkage analysis using drink24 and pakyrs as two correlated behavioral measures. Previous linkage studies have investigated these measures individually [5,6], but currently no study has considered them in a bivariate analysis. Bivariate quantitative linkage analyses have been shown to identify genes with small effects where these genes may be missed with univariate analyses. However, these multivariate linkage analyses are computationally intensive as the number of traits used in the analysis increases. The proposed screening test combines univariate linkage results to determine whether a bivariate linkage analysis might be beneficial.

## Methods

### Data description

The COGA data consisted of 143 extended families, a mixture of large and small families, with 1,350 family members with clinical and demographic data. Because these families consisted of different ethnicities, we analyzed the families that were white, non-Hispanic (WNH). A family was considered WNH if 75% of the reported ethnicity in the family was WNH; thus, our analyses were performed on 115 extended families. The phenotypes selected for the analysis were drink24 and pakyrs. Because drink24 and pakyrs measures have skewed distributions, a square root transformation (sqrt) was applied in both measures to normalize the distribution.

### Genetic markers

The microsatellite markers and the Illumina SNPs were each used for our analyses. For the Illumina SNPs, we removed SNPs that were in linkage disequilibrium (LD) with another SNP. We based our criteria for LD using r^2^, and the cut-off value of 0.4, which from our experience removed the effects of LD without a great loss of information. After dropping the SNPs in LD, a total of 350, 258, and 161 SNPs on chromosomes 1, 4, and 9, respectively, were used in our analyses. Multipoint identity-by-descent (MIBD) sharing among pairs of relatives was calculated for microsatellite and SNP markers using the SIMWALK2 software program [7]

### Quantitative trait linkage analysis

For the quantitative linkage analysis, we used the locally developed SPLUS *multic *library. This is a new library based on the C++ multic program from ACT [8]. For the analysis, we performed univariate and bivariate quantitative linkage analysis using a variance components (VC) approach. The details about univariate and multivariate quantitative linkage analysis are described in Amos [9] and de Andrade and Amos [10]. Sqrt(pakyrs) and sqrt(drink24) were adjusted for age and sex in the linkage analyses.

To test for genetic linkage, a likelihood ratio test (LRT) was applied. Under the null hypothesis, the linked gene parameter(s) is (are) restricted to equal 0. The distributions of the univariate and bivariate linkage tests are a mixture of 1/2 χ_0_^2 ^and 1/2 χ_1_^2^, and a mixture of 1/4 χ_0_^2^, 1/2 χ_1_^2^, and 1/4 χ_3_^2^, respectively [11]. In the univariate linkage analyses, we considered multipoint maximum LOD scores (MLS) ≥ 3.00 as statistically significant evidence of linkage, ≥ 2.00 as suggestive evidence, and ≥ 1.30 as tentative evidence of linkage [12]. These MLS thresholds correspond to *p*-values of 0.0001, 0.001, and 0.007, respectively. To achieve levels of statistical significance in the bivariate linkage analysis comparable to the univariate thresholds, we calculated the threshold using a mixture of 1/4 χ_0_^2^, 1/2 χ_1_^2^, and 1/4 χ_3_^2^. This calculation provided MLS ≥ 4.00 as statistically significant evidence of linkage (i.e., *p *≤ 0.0001), ≥ 2.87 as suggestive evidence (i.e., *p *≤ 0.001), and ≥ 2.06 as tentative evidence of linkage (*p *≤ 0.007). We inferred evidence of chromosomal regions with pleiotropic effects when the bivariate MLS met the criteria for at least tentative evidence of linkage and its nominal *p*-value was less than the univariate maxima at the same location.

### Screening test

Let us assume *k *quantitative traits are represented by **Y**_1_, **Y**_2_, ..., **Y**_k_. For each trait a genome-wide scanning linkage analysis is performed using the VC quantitative trait approach. For each trait *i*, and genomic position *j*, the quantitative trait locus (QTL) variance component estimate (σ^2^_ij_) is estimated with its standard error. Our hypothesis for the proposed screening test is: if there is a gene with pleiotropic effects, its QTL VC should be incremented in an additive manner using combinations of correlated traits by simply adding its respective univariate QTL VC. Let σ^2^_ijk _be the QTL VC for trait *i*, position *j *on chromosome *k*. The null hypothesis is that there is no pleiotropic effect at position *j *on chromosome *k*, i.e., H_0_:  = 0 ∀ *i*, *j*, *k*, which is equivalent to H_0_: . The alternative hypothesis is H_1_: σ^2^_*ijk *_> 0 for any *i*, *j*, *k*. The test statistic will be , where  is the maximum likelihood estimator (MLE) of σ^2^_*ijk*_. Under H_0_, E(σ^2^_*ijk*_) = 0, ∀ *i*, *j*, *k*, S_*ijk *_~ 1/2 N (0, 1). By assuming the S_*ijk *_values are independent, , where T is the number of traits. Consequently by squaring and standardizing T_*jk*_, [11].

## Results

**Figure 1:**
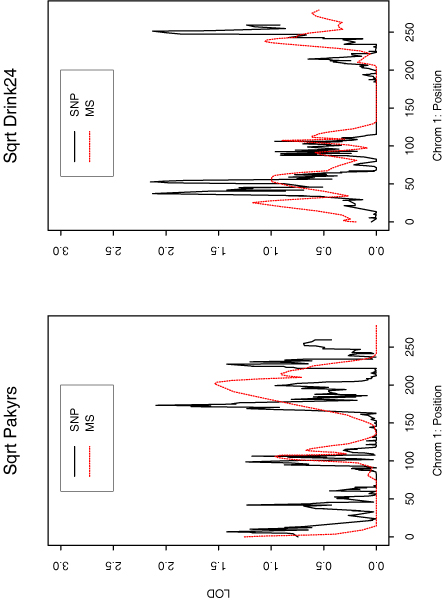
Comparison between SNP and microsatellite (MS) markers on chromosome 1 for the sqrt(pakyrs) and sqrt(drink24).

Genome-wide linkage analyses were performed for all autosomal chromosomes using microsatellite markers, and only on three chromosomes (1, 4, and 9) using Illumina SNPs. These three chromosomes were selected because they contain regions of interest based on previous studies [1]. For microsatellite markers, the univariate linkage analyses of sqrt(pakyrs) demonstrated tentative evidence of linkage on chromosomes 1 (LOD = 1.53, *p *= 0.004, 201 cM) and 8 (LOD = 1.87, *p *= 0.0017, 14 cM), and suggestive evidence of linkage on chromosomes 2 (LOD = 2.02, *p *= 0.0011, 144 cM) and 14 (LOD = 2.46, *p *= 0.00038, 107 cM). For sqrt(drink24), there was tentative evidence of linkage on chromosome 10 (LOD = 1.37, *p *= 0.006, 159 cM) and suggestive evidence of linkage on chromosome 13 (LOD = 2.19, *p *= 0.0007, 63 cM). For SNP markers, we observed an increase in the LOD scores compared to microsatellite markers on chromosome 1 for sqrt(pakyrs) (SNP LOD = 2.10, *p *= 0.0009,173 cM) and for sqrt(drink24) (SNP LOD = 2.15, *p *= 0.0008, 52 cM; microsatellite LOD = 1.18, *p *= 0.0099, 52 cM), and on chromosome 4 for sqrt(drink24) (SNP LOD = 1.68, *p *= 0.0027, 121 cM; microsatellite LOD = 0.98, *p *= 0.016, 43 cM). Figure 1 shows a direct comparison of microsatellite and SNP results for chromosome 1 using each trait separately.

**Figure 2:**
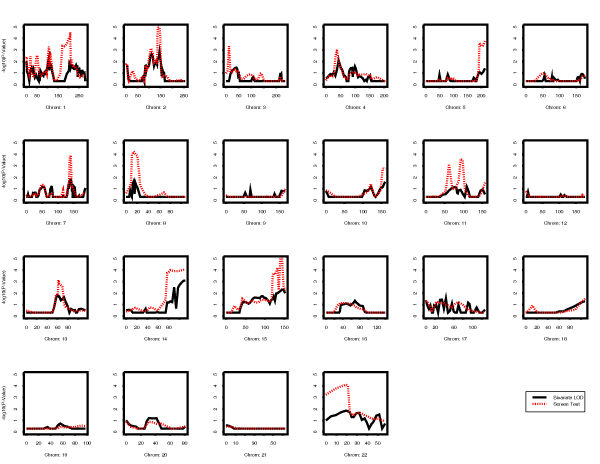
Multipoint results of bivariate linkage analysis and the screening test for sqrt(pakyrs) and sqrt(drink24).

The phenotypic correlation between sqrt(pakyrs) and sqrt(drink24) was 0.38 and the genetic correlation was 0.70, indicating that these two measures shared common genes. For the bivariate analyses no significant evidence of genes with pleiotropic effects on sqrt(pakyrs) and sqrt(drink24) was observed using either microsatellite or SNP markers. Our proposed screening test detected some genomic regions of interest, although not at the bivariate level of significance. Figure 2 depicts the results of the bivariate genome-wide linkage analysis using sqrt(pakyrs) and sqrt(drink24) and the screening test. The screening test detected several regions in which a bivariate analysis may be appropriate to use; however, in these regions only one of the two traits showed evidence of linkage. For instance, the results of the screening test on chromosomes 1 and 2 are due to the univariate linkage results of sqrt(pakyrs) and not due to the bivariate results (data not shown).

## Discussion

In our analyses using microsatellite markers, tentative and suggestive evidence of linkage were found on chromosomes 1, 2, 8, and 14 for sqrt(pakyrs) and on chromosomes 10 and 13 for sqrt(drink24). Bergen et al. identified several regions for sqrt(pakyrs) in the COGA sample, among chromosomes 2 (~10 cM) and 14 (~68 cM) [13]. Straub et al. identified several linkage regions for nicotine dependence in a sample from Christchurch, New Zealand within chromosome 2 (~150 cM, LOD = 1.5) [14]. Saccone et al. identified a susceptibility locus on chromosome 4 (~120 cM, LOD = 3.5) for drink24 [5]. In our analysis using SNP markers, we observed an increase in the LOD scores and suggestive evidence of linkage on chromosomes 1 and 4 for sqrt(drink24) that was not observed using microsatellite markers. No evidence of a pleiotropic effect was found between sqrt(pakyrs) and sqrt(drink24). Our screening test is a computationally time-saving approach that can be used to determine which regions should be analyzed using a multivariate approach. However, significant results of the screening test may be misleading because the results may be driven by only one trait rather than several traits. Thus, careful evaluation of the univariate linkage results and the screening test is necessary.

During our analyses several difficulties arose when SNPs were used in quantitative trait linkage analysis. First, the only software that could specifically handle pedigrees of large size was SIMWALK2 [8]; however, it was computationally intensive to estimate the MIBDs. Second, in order to calculate the MIBD for 350 SNPS on chromosome 1, we had to break the 350 SNPs into 10 groups of 35 SNPs and then combine the results of the linkage analyses.

## Conclusion

We observed evidence of linkage on chromosome 4 for alcohol consumption using SNPs; this linked region was in the same region previously identified by Saccone et al. [5]. Furthermore, using SNPs, we also observed several suggestive regions for linkage to sqrt(pakyrs) and sqrt(drink24) not previously identified. The proposed screening test for multivariate quantitative trait linkage analysis also showed its potential application in this data. Our experience using large extended families and many SNPs suggest that software limitations are an issue when contemplating genome-wide linkage scans.

## Abbreviations

COGA: Collaborative Study on the Genetics of Alcoholism

LD: Linkage disequilibrium

LRT: Likelihood ratio test

MIBD: Multipoint identity-by-descent

MLE: Maximum likelihood estimator

MLS: Maximum LOD score

QTL: Quantitative trait locus

SNP: Single-nucleotide polymorphism

VC: Variance components

WNH: White, non-Hispanic

## Authors' contributions

All the authors contributed equally in the analysis and in the preparation of the manuscript. All authors read and approved the final manuscript.
